# Depletion of Intestinal Microbiome Partially Rescues Bone Loss in Sickle Cell Disease Male Mice

**DOI:** 10.1038/s41598-019-45270-4

**Published:** 2019-06-17

**Authors:** Sara Tavakoli, Liping Xiao

**Affiliations:** 1 Department of Medicine, UConn Health, Farmington, CT 06030 USA; 2 Department of Psychiatry, UConn Health, Farmington, CT 06030 USA

**Keywords:** Sickle cell disease, Mechanisms of disease

## Abstract

Osteoporosis or osteopenia are common clinical manifestations of sickle cell disease (SCD) with unclear mechanisms. Since senescence of circulating neutrophil can be modulated by signals derived from intestinal microbiome and neutrophils are abundant in bone marrow and can regulate osteoblasts and osteoclasts, we examined whether gut microbiome contributes to bone loss in SCD mice. SCD and their littermates control mice were treated with antibiotics to deplete gut microbiome. At the end of 7 weeks treatment, serum was collected for biochemistry marker measurements. Bone mass and remodeling were evaluated by dual beam X-ray absorptiometry, micro-computed tomography, and histomorphometry. Bone-related genes in tibia and barrier marker genes in the small intestine were analyzed by quantitative PCR. Antibiotic treatment rescued increased intestinal inflammatory cytokine marker genes (*Tnfα*, *IL17*, *Ifnγ*) expression, rescued decreased intestinal barrier marker genes (*claudin 3* and *claudin 15*) expression, and rescued increased serum cytokines (IFNγ, IL27, IL10) in SCD mice. Antibiotic significantly improved decreased bone mass in SCD mice mainly through enhanced osteoblast function and increased osteoblast-related genes (*Runx2* and *Igf1*) expression in SCD mice. Our findings support that increased bacteria load augments antigenic load traversing the impaired intestinal barrier through inflammation, leading to increased inflammatory cytokines, impaired osteoblast function, and bone loss in SCD mice.

## Introduction

Sickle cell disease (SCD) is the most popular genetic blood disease^1^ caused by a single nucleotide mutation of β-hemoglobin gene that turns normal round red blood cells into sickle cells that can get stuck in blood vessels and block blood flow. Although SCD is a hemoglobin disorder, it is associated with an increased incidence of bone complications^2^. Bone involvement includes joint and bone pain, bone infarcts, osteomyelitis, arthritis, avascular necrosis, vertebral collapse, osteopenia, osteoporosis, and pathological fracture^3,4^. However, the mechanisms of bone loss in SCD are still poorly understood due to the complex multifactorial pathophysiology of sickle cell bone disease.

Currently, effective treatment options for sickle cell bone disease are lacking and there are no consensus guidelines for the use of well-established anti-osteoporotic therapies in SCD patients^2^. Potential adverse effects of these reagents could limit their use in SCD patients. Bone, joint, and muscle pain as well as osteonecrosis of the jaw, may occur at any point after patients begin taking a bisphosphonate^5,6^, which could potentially worsen acute and chronic pain, and osteonecrosis often seen in SCD patients. The anabolic effect of PTH1-34 decreases with time and repeated treatment^7^ and high PTH levels are often seen in SCD patients^8,9^. Therefore, there is a need to seek new therapies for sickle cell bone disease.

Neutrophil senescence mediated by signals derived from intestinal microbiome regulates chronic inflammation-related organ damage in SCD^10^. Depletion of intestinal microbiome decreased neutrophil senescence in circulation and partially rescued the liver and spleen damage in SCD mice^10^. We recently reported that bone marrow neutrophil senescence was higher in Townes SCD mice compared with their littermates control (Ctrl)^11^. Administration of antibiotics (Abx) to SCD mice inhibits the bone marrow neutrophil senescence and rescues the impaired osteoblast function in osteoblasts co-cultured with bone marrow neutrophils^11^. Bone marrow is the site of neutrophil production and the site of clearance of senescent neutrophils from the circulation^12,13^. Since bone marrow neutrophils can modulate osteoblast and osteoclast function^14^, and inflammatory-induced bone marrow neutrophilia decrease osteoblast number^15,16^, we examined whether there are changes in intestinal microbiome and whether this is related to bone loss in SCD mice *in vivo*.

In this report, we administered broad-spectrum Abx to healthy Ctrl and SCD male mice to deplete gut microbiome and analyzed bone phenotypes *in vivo*. We demonstrated that SCD mice had increased gut bacterial load, inflammation, and intestinal permeability marker genes, as well as reduced bone mass and impaired osteoblast functions. Depletion of gut microbiome partially rescued bone loss in SCD mice. These suggest that microbiome modification might be a potential adjuvant therapy for sickle cell bone disease.

## Results

### Microbiome depletion with Abx improved splenomegaly, rescued increased intestinal/serum inflammatory cytokines, and rescued decreased barrier marker genes in SCD mice

**Figure 1:**
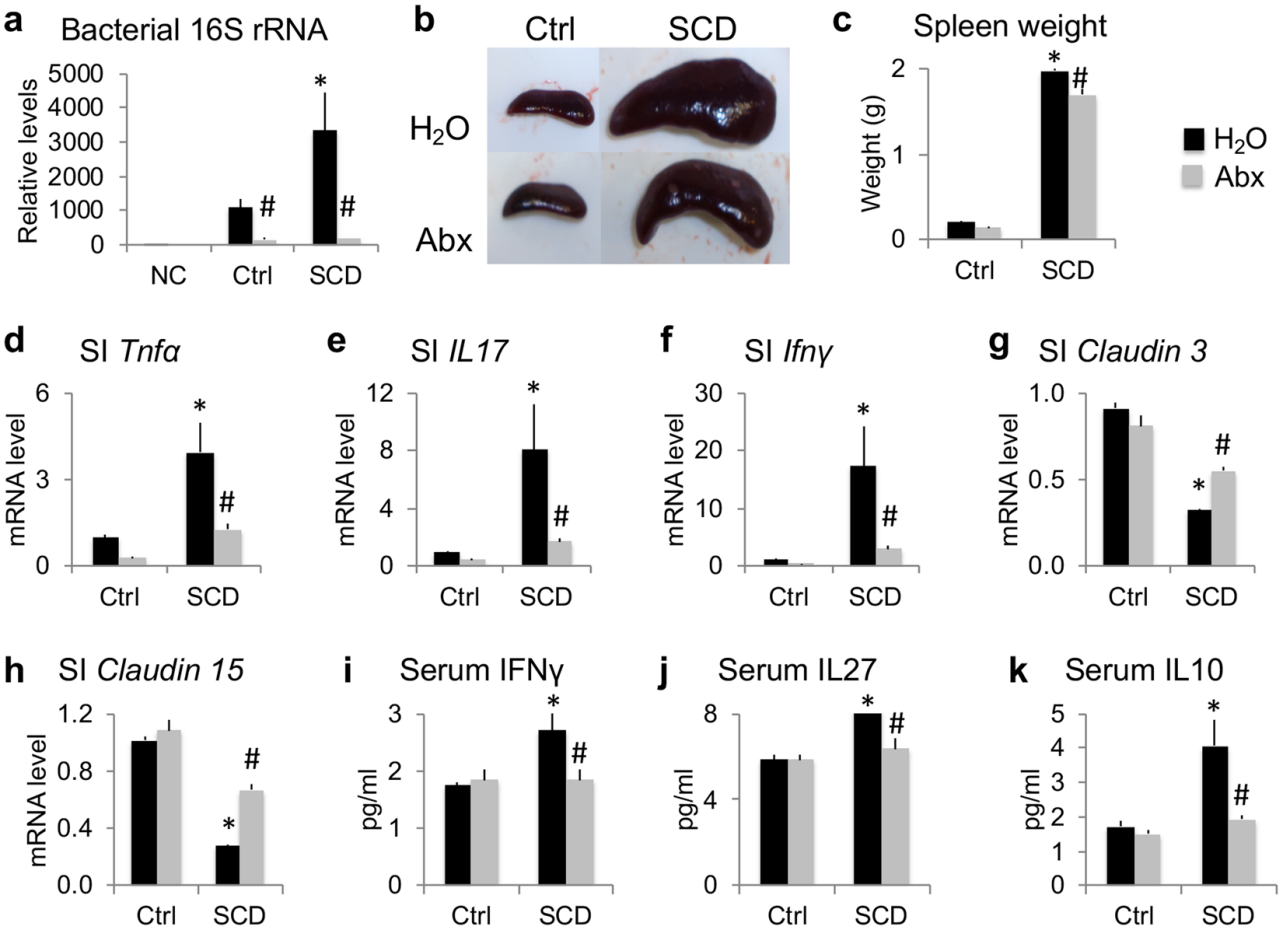
Microbiome depletion with Abx improves splenomegaly, rescues increased intestinal/serum inflammatory cytokines, and rescues decreased barrier marker genes in SCD male mice. (**a**) Increased gut bacteria load in SCD mice and bacterial depletion after Abx treatment in both Ctrl and SCD mice. NC: negative control, same processing but without feces. n = 4 mice/group. (**b**) Representative images of the spleen. (**c**) Spleen weight. n = 4 mice/group. (**d**–**h**) RT-qPCR of RNA from the middle 20 mm of the small intestine (SI) analyzed for (**d**) *Tnfα*, (**e**) *Il17*, (**f**) *Ifnγ*, (**g**) *claudin 3*, and (**h**) *claudin 15*. n = 4 mice/group. (**i**–**k**) Luminex Assays of serum (**i**) INFγ, (**j**) IL27, and (**k**) IL10. n = 4 mice/group. Data are mean ± SE. *p < 0.05 compared with Ctrl-H_2_O; ^#^*p* < 0.05 compared with corresponding H_2_O group by two-way ANOVA.

Ctrl and SCD male mice were caged individually (housed separately) at 4 months of age and were assigned to H_2_O or Abx groups randomly. After 7 weeks treatment, in contrast to Ctrl-H_2_O group, analysis of 16S rRNA gene copy number showed that gut bacterial load was significantly higher in the SCD mice treated with H_2_O group compared to Ctrl mice treated with H_2_O. Abx administration significantly decreased the total bacterial load in both Ctrl and SCD mice (Fig. 1a), and the splenomegaly (as evidenced by increased spleen size and weight) in SCD mice (Fig. 1b–c).

Since SCD is associated with chronic inflammation^10,17,18^, and inflammation in the intestine can decrease intestinal barrier integrity and increase the antigenic load traversing the intestinal barrier^19^, we measured mRNA levels of inflammatory cytokines *Tnfα*, *IL17*, and *Ifnγ*, and gap junction protein marker genes, *claudin 3* and *claudin 15* (which have been shown to modulate intestinal barrier integrity^19^), in small intestine of 6-month-old single-housed male mice. As shown in Fig. 1d–f, *Tnfα*, *IL17*, and *Ifnγ* mRNA were significantly increased in the small intestine of SCD mice and were significantly reduced with Abx treatment. *Claudin 3* and *claudin 15* mRNA levels were significantly decreased in SCD mice compared with Ctrl and were significantly increased with Abx treatment. Serum levels of inflammatory cytokines IFNγ, IL27, IL10, were higher in the SCD mice treated with H_2_O compared to the Ctrl mice treated with H_2_O group and were significantly decreased with Abx treatment (Fig. 1i–k).

### Microbiome depletion with Abx improved decreased body weight, hindlimb BMD, and BMC in SCD male mice

**Figure 2:**
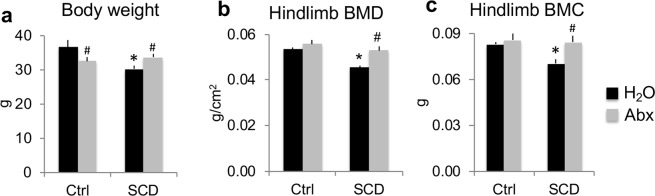
Microbiome depletion with Abx improved decreased body weight, hindlimb BMD, and BMC in SCD male mice. (**a**) Body weight. (**b**) Hindlimb BMD and (**c**) BMC were significantly decreased in SCD-H_2_O mice compared with Ctrl-H_2_O mice and Abx treatment significantly increased hindlimb BMD and BMC in SCD mice. n = 4 mice/group. Data are mean ± SE. *p < 0.05 compared with Ctrl-H_2_O; ^#^*p* < 0.05 compared with corresponding H_2_O group by two-way ANOVA.

At the end of treatment at 6 months of age, body weight was lower in the SCD-H_2_O group compared with the Ctrl-H_2_O group, and Abx treatment significantly decreased body weight in Ctrl mice, but increased body weight in SCD mice (Fig. 2a). To determine whether Abx treatment regulates BMD and bone mineral content (BMC) in SCD mice, we measured BMD and BMC *in vivo* at the end of the 7-week treatment. Hindlimb BMD and BMC were significantly decreased in the SCD-H_2_O group compared with Ctrl-H_2_O; these phenotypes were rescued with Abx treatment (Fig. 2b,c). These data suggest that microbiome are responsible for decreased BMD and BMC in SCD mice.

### Micro-computed tomography (μCT) analysis of femurs showed Abx-improved bone mass was more pronounced in SCD vs. Ctrl mice

**Figure 3:**
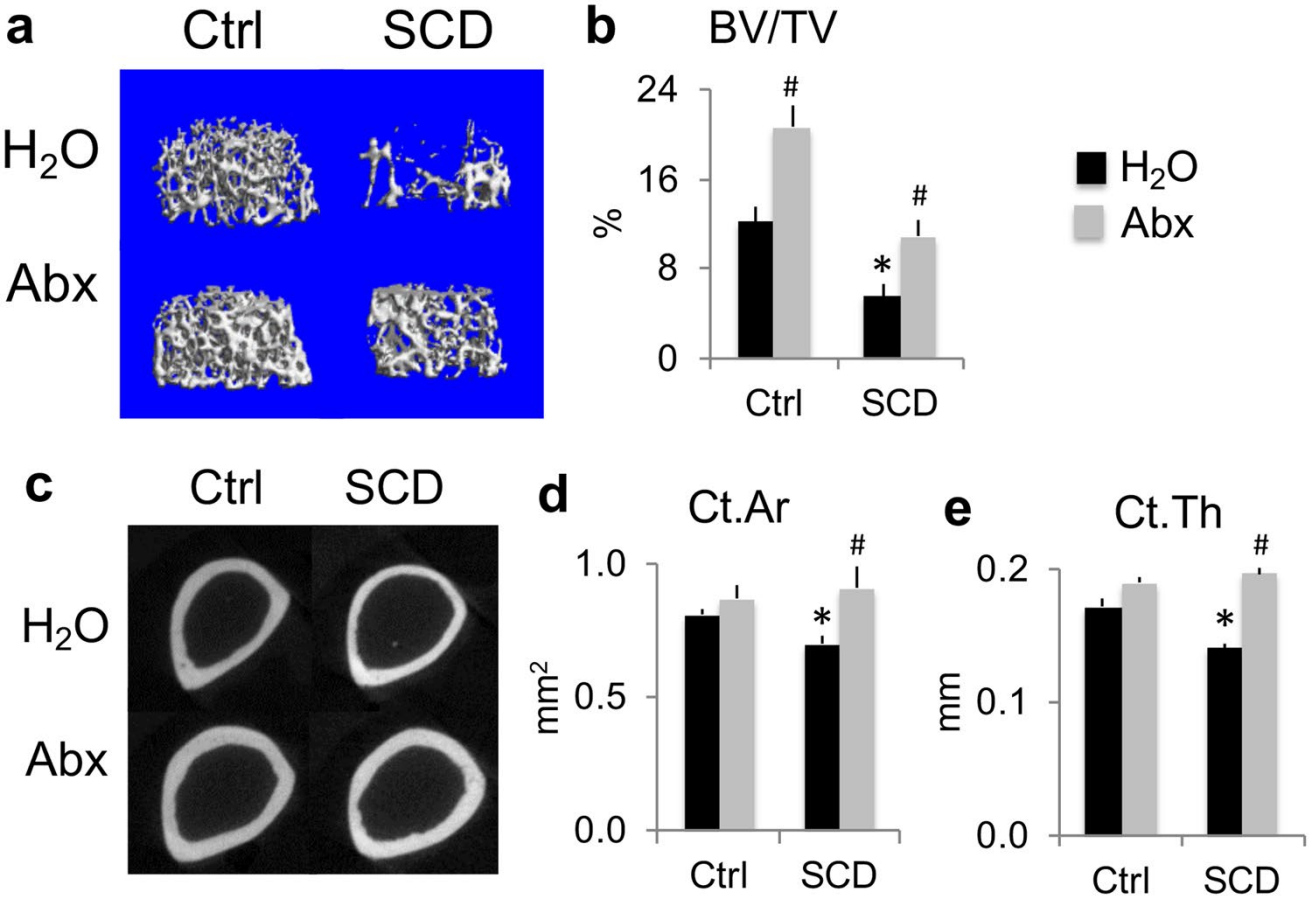
Several bone phenotypes in SCD male mice were rescued by Abx as determined by μCT analysis of femurs. (**a**) Representative μCT images of distal femur metaphysis cancellous bone. (**b**) Quantification of metaphysis bone volume/total volume (BV/TV). (**c**) Representative μCT images of mid-diaphysis cortical bone of femur. Quantification of (**d**) mid-diaphysis cortical area (Ct.Ar), (**e**) cortical thickness (Ct.Th). n = 4 mice/group. Data are mean ± SE. *p < 0.05 compared with Ctrl-H_2_O; ^#^*p* < 0.05 compared with corresponding H_2_O group by two-way ANOVA.

To characterize the effects of Abx on bone structure, μCT was performed on femurs at the end of 7 weeks of treatment. μCT analysis of distal femur metaphyseal trabecular bone showed SCD-H_2_O mice have a lower initial bone volume/total volume (BV/TV) than Ctrl-H_2_O, and BV/TV was restored to Ctrl-H_2_O levels after Abx treatment (Fig. 3a,b). Abx treatment significantly increased BV/TV by 70% in Ctrl and 99% in SCD mice. μCT analysis of diaphyseal cortex showed significant decreases in cortical area and cortical thickness in the SCD mice treated with H_2_O compared to Ctrl mice treated with H_2_O that were improved after Abx administration (Fig. 3c–f). These data suggest that depletion of gut microbiome is sufficient to drive these bone phenotypic changes, with more pronounced effects in SCD mice; the changes in cortical bone of SCD mice were much greater than in Ctrl mice.

### Bone histomorphometry of femur of Ctrl and SCD male mice with and without Abx treatment

**Figure 4:**
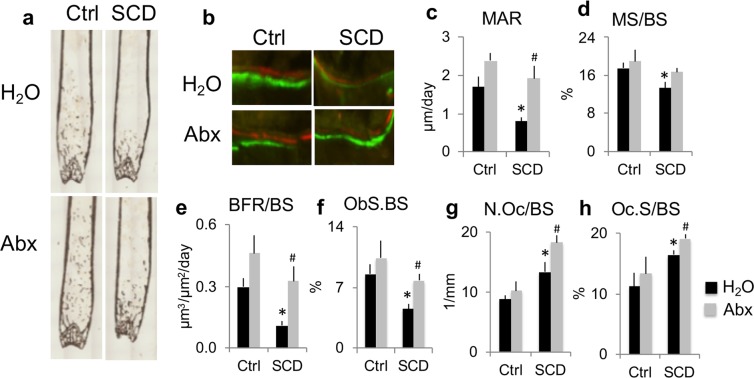
Microbiome depletion with Abx rescued several bone phenotypes in SCD male mice as determined by bone histomorphometry. (**a**) von Kossa staining shows decreased trabeculae in SCD-H_2_O group that was partially rescued with Abx treatment. (**b**) Calcein and Xylenol orange labeling shows decreased double-label distance in the SCD-H_2_O group compared with the Ctrl-H_2_O group, that was partially rescued with Abx treatment. (**c**) Mineral apposition rate (MAR), (**d**) mineral surface/bone surface (MS/BS), (**e**) bone formation rate/bone surface (BFR/BS), (**f**) osteoblast surface per bone surface (ObS/BS), (**g**) osteoclast number/bone surface (N.Oc/BS), (**h**) osteoclast surface/bone surface (OcS/BS). n = 4 mice/group. Data are mean ± SE. *p < 0.05 compared with Ctrl-H_2_O; ^#^*p* < 0.05 compared with corresponding H_2_O group by two-way ANOVA.

To examine the effects of Abx on bone structure in more detail, bone histomorphometry was performed on femurs harvested after 7 weeks of treatment. Von Kossa staining showed decreased trabeculae in SCD-H_2_O mice compared with Ctrl-H_2_O that was partially rescued with Abx treatment (Fig. 4a). To examine osteoblast function at the cellular level, dynamic bone histomorphometry was performed. Dynamic histomorphometry revealed decreased double labeling distance and mineral apposition rate (reflecting the activity of individual teams of osteoblasts) in the SCD-H_2_O group compared with Ctrl-H_2_O, and these parameters were rescued by Abx treatment (Fig. 4b,c). Mineral surface/bone surface was decreased in the SCD-H_2_O group compared with Ctrl-H_2_O (Fig. 4d). Decreased bone formation rate and osteoblast surface/bone surface in the SCD-H_2_O group were partially rescued with Abx treatment (Fig. 4e,f). Masson’s Trichrome staining did not show osteoid accumulation in any group (data not shown). Osteoclast number/bone surface and osteoclast surface/bone surface were significantly higher in SCD mice treated with H_2_O compared to Ctrl mice treated with H_2_O, and were further increased with Abx treatment (Fig. 4g,h). These data suggest that Abx significantly improved bone mass in SCD mice mainly through enhanced osteoblast function.

### Bone-related gene expression in tibia of Ctrl and SCD male mice with and without Abx treatment

**Figure 5:**
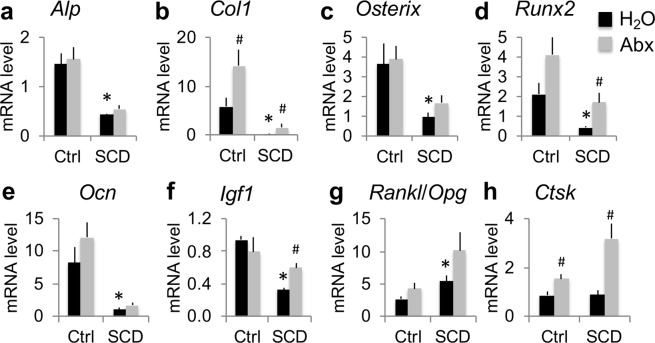
Several bone-related genes in flushed tibiae were reduced in SCD male mice and partially rescued with Abx treatment. RT-qPCR of flushed tibiae for (**a**) *ALP*, (**b**) *Col1*, (**c**) *Osterix*, (**d**) *Runx2*, (**e**) *Ocn*, (**f**) *Igf1*, (**g**) *Rankl*/*Opg*, (**h**) *Ctsk*. n = 4 mice/group. Data are mean ± SE. *p < 0.05 compared with Ctrl-H_2_O p < 0.05; ^#^*p* < 0.05 compared with corresponding H_2_O group by two-way ANOVA.

To determine whether increased bone volume in Abx-treated SCD mice is associated with enhanced expression of genes involved in bone homeostasis, mRNA was extracted from the flushed tibiae of H_2_O- or Abx-treated Ctrl and SCD mice at the end of 7 weeks treatment. RT-qPCR analysis revealed a significant decrease in osteoblast gene markers (Fig. 5), including *alkaline phosphatase* (*Alp*), *type 1 collagen* (*Col1*), *Osterix*, *runx-related transcription factor 2* (*Runx2*; a transcription factor that is important for osteoblast differentiation), *osteocalcin* (*Ocn*, a terminal osteoblast marker gene), and *insulin-like growth factor 1* (*Igf1*), in SCD-H_2_O compared with Ctrl-H_2_O. Abx treatment significantly increased *Col1* expression in both genotypes but significantly increased *Runx2* and *Igf1* mRNA expression only in SCD mice. Also shown in Fig. 5, the assessment of osteoclast-related genes revealed a significantly increased *Rankl*/*Opg* ratio, an important determinant of osteoclast formation, in SCD-H_2_O compared with Ctrl-H_2_O. There was no difference in late osteoclast marker gene *Cathepsin K* (*Ctsk*) mRNA expression between Ctrl-H_2_O and SCD-H_2_O. Abx treatment significantly increased *Ctsk* mRNA expression in both genotypes.

### Serum biochemistry markers in Ctrl and SCD male mice with and without Abx treatment

**Figure 6:**
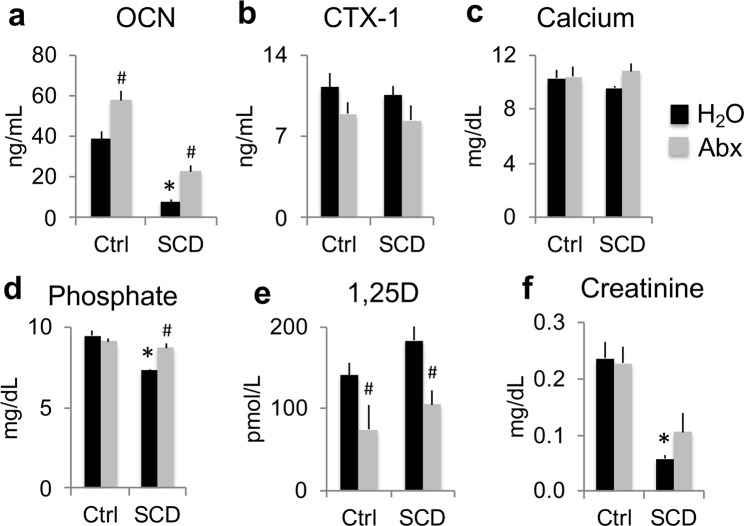
Serum biochemistry markers were differentially affected in SCD male mice and partially rescued by Abx treatment. Serum measurements of (**a**) OCN, (**b**) CTX1, (**c**) calcium, (**d**) phosphate, (**e**) 1,25-dihydroxy vitamin D3 (1,25D) and (**f**) creatinine. n = 4 mice/group. Data are mean ± SE. *p < 0.05 compared with Ctrl-H_2_O p < 0.05; ^#^*p* < 0.05 compared with corresponding H_2_O group by two-way ANOVA.

Serum bone formation marker OCN protein level was significantly decreased in the SCD-H_2_O group compared with the Ctrl-H_2_O group. Abx treatment significantly increased serum OCN by 48% in Ctrl mice, and 198% in SCD mice (Fig. 6a). Serum bone resorption marker C terminal telopeptides of type l collagen (CTX1) protein was similar between Ctrl-H_2_O and SCD-H_2_O groups. There were no significant differences in serum CTX1 protein levels after Abx treatment in both genotypes (Fig. 6b). To determine whether Abx treatment modulates calcium/phosphate homeostasis in Ctrl and SCD mice, serum calcium and phosphate were measured at the end of the 7-week study. As shown in Fig. 6c, there was no difference in serum calcium among groups. Serum phosphate level was significantly lower in the SCD mice treated with H_2_O compared to the Ctrl mice treated with H_2_O and was corrected with Abx administration (Fig. 6d). Since 1,25-dihydroxy vitamin D3 (1,25D) plays an important role in regulating phosphate homeostasis, we measured its levels in serum. As shown in Fig. 6e, there was no significant difference in serum 1,25D between Ctrl-H_2_O and SCD-H_2_O groups. Abx treatment significantly decreased 1,25D levels in both Ctrl and SCD mice. Serum creatinine was significantly decreased in the SCD-H_2_O group compared with the Ctrl-H_2_O group (Fig. 6f).

## Discussion

In the present study using the Townes SCD mouse model that phenocopies human SCD, we demonstrated a significant reduction in BMD and BMC in SCD male mice compared to healthy Ctrl mice. We also demonstrated that SCD mice exhibit increases in gut bacterial load, inflammation, and intestinal permeability, as well as a reduction in bone mass and impaired osteoblast function, most of which were rescued after depletion of intestinal microbiome with broad-spectrum Abx administration.

Since the research^10^ indicated that depletion of microbiome with Abx dramatically improves the pathogenesis and inflammation-related spleen and liver damage in Berkeley SCD mice, we tested whether there were similar changes in Townes SCD mice, which were used in the current study. We found that Abx treatment significantly reduced splenomegaly in SCD mice. Other previous studies showed that SCD is associated with chronic inflammation^10,17,18,20^ and inflammation in the intestine can decrease intestinal barrier integrity and increase the antigenic load traversing the intestinal barrier^19^. Consistent with these observations, we found *Tnfα*, *IL17*, and *Ifnγ* mRNA were significantly increased in small intestines of SCD mice; and *claudin 3* and *claudin 15* mRNA were significantly decreased in SCD mice compared with Ctrl, suggesting increased intestinal permeability; these gene expression changes were partially rescued with Abx treatment.

Previous studies showed that anti-inflammatory cytokines and pro-inflammatory cytokines are elevated in SCD patients compared with controls^21^. Elevation of anti-inflammatory cytokines in response to pro-inflammatory cytokines is sufficient in preventing further cellular or tissue damage but not potent enough to prevent inflammation^21^. Consistent with this, our findings from this study show that anti-inflammatory cytokines (IL-27 and IL-10) as well as pro-inflammatory cytokines (IFNγ) were markedly up-regulated in SCD mice and were rescued with Abx treatment. Leukocytes, including neutrophils, are important sources of cytokines^22^. There are significantly increased neutrophils and aged neutrophils in bone marrow of SCD mice^11^, senescent neutrophils upregulated several pathways that were also enhanced during neutrophil activation^10^, therefore elevated levels of aged neutrophils of SCD mice may have partly contributed to the elevated circulating levels of pro-inflammatory cytokines.

A four-drug cocktail of antibiotics (ampicillin, vancomycin, neomycin, and metronidazole) in drinking water is commonly used to deplete the gut microbiome in mouse models^10^. Some groups add low caloric aspartame-based sweetener in the cocktail in an attempt to mask the taste of the antibiotics. Since it is known that aspartame-based sweetener impacts bone metabolism^23^, we did not add sweetener to the Abx cocktail. Ctrl animals fed Abx did not show any sign of dehydration, such as decreased skin turgor and hard stools, although body weight gain was much less in the Ctrl-Abx group than in the Ctrl-H_2_O group over the 7-week treatment (0.31% vs. 7.26%). Interestingly, in contrast to a 3.80% weight gain in the SCD-H_2_O group over the 7-week treatment, body weight gain was much higher (9.36%) in SCD-Abx mice. This suggests that Abx might be beneficial in SCD mice.

The human microbiome has been shown to influence a number of chronic conditions associated with impaired bone quality^24,25^. However, conflicting results of the microbiome regulating bone health have been reported^19,26–32^. Difference in mouse genetic background, sex, age, inherent differences in microbial communities that is specific to location^33^ and disease conditions could account for the differences observed. In addition, altering microbiome composition, through methods such as treating mice with pathogenic bacteria^34^ or probiotics^35,36^, can decrease or increase bone density, respectively. Recently intestinal microbiome analysis revealed dysbiosis in humans with SCD^37^. Our unpublished data also showed the changes in the composition of the gut microbiome in SCD mice with a marked increase in potentially pathogenic bacteria families. Therefore, increased bone mass in SCD mice after Abx treatment could be due to depletion of pathogenic bacteria and/or decrease on bacterial load. Our μCT results demonstrated significant alterations in structural parameters in both trabecular and cortical compartments of femurs in SCD mice compared with Ctrl mice. Abx treatment significantly increased bone volume/total volume in both Ctrl and SCD mice. However, the percentage increase was more pronounced in SCD mice. Interestingly, Abx increased cortical area and cortical thickness only in SCD mice. In combination with BMD and BMC results, these data suggest that depletion of gut microbiome with Abx have more pronounced effects on bone in SCD mice than Ctrl mice.

Bone histomorphometry of femur trabeculae further demonstrated reduced bone mass in SCD mice and their response to Abx treatment at the cellular level. There were significant reductions in new bone formation parameters, including double-labeling distance, mineral apposition rate, mineral surface/bone surface, and bone formation rate in SCD-H_2_O compared with Ctrl-H_2_O mice. This is consistent with the impaired osteoblast function we reported in SCD female mice^38^. Abx treatment significantly increased mineral apposition rate and bone formation rate only in SCD mice. These results indicate that increased bone mass after Abx treatment in SCD mice is due in part to increased new bone formation. The reduction in osteoblast-related genes in SCD male mice is consistent with our reported studies in SCD female mice^38^ that reflect impaired osteoblast function. Abx treatment significantly increased *Runx2* and *Igf1* mRNA expression only in SCD mice and increased serum OCN protein more in SCD mice than Ctrl mice, which could explain why there is a more pronounced bone formation in SCD mice than Ctrl mice after Abx treatment.

In our present study, histomorphometry of femur trabeculae showed increased osteoclast number in SCD mice, which is consistent with our observation in SCD female mice^38^. Although the osteoclast marker gene *Rankl*/*Opg* ratio was significantly higher in SCD mice treated with H_2_O mice compared to Ctrl mice treated with H_2_O, mature osteoclast marker gene, *Ctsk*, and serum CTX1 were not increased in SCD-H_2_O mice. This is consistent with a previous study that found increased osteoclast and no change in CTX1 in SCD mice under normoxic conditions^39^. It should be noted that our published *in vitro* study showed that bone marrow macrophages cultured from SCD and Ctrl female mice formed a similar number of pits and resorption areas on bone slices^38^. This suggests that there is no intrinsic osteoclast defect in SCD mice.

Our current studies showed that there was no difference in serum calcium between Ctrl and SCD mice, and there was no difference in serum calcium before and after Abx treatment in both Ctrl and SCD mice, indicating that depletion of gut microbiome did not affect gut calcium absorption in either genotype. Serum phosphate levels have been reported as high^40^, normal^41^, or low^42^ in SCD subjects. In the current study, we found decreased serum phosphate that was rescued with Abx treatment in SCD mice compared with Ctrl mice. Vitamin D deficiency is also prevalent in SCD subjects^43,44^. Studies showed that bone loss in SCD may be due in part to vitamin D deficiency that contributes to reduced bone mass due to osteomalacia resulting from impaired matrix mineralization^44,45^. However, in the current study, we did not observe osteoid accumulation by Masson’s Trichrome staining (data not shown), and we did not observe differences in serum 1,25D levels between Ctrl-H_2_O and SCD-H_2_O mice. This may be due to the mice being maintained on a standard diet that contained fixed amounts of vitamin D. Decreased serum 1,25D in both Ctrl and SCD mice after Abx treatment may reflect disrupted vitamin D absorption in the small intestine due to depletion of gut bacteria. In this study, we observed low serum creatinine in SCD mice that may reflect lower muscle mass due to muscle wasting and low protein stores, which is often observed in SCD subjects^46^.

The bone marrow is a large reservoir of mature neutrophils and is the major organ to which senescent neutrophils home for clearance^12,13^. The microbiome drives neutrophil ageing through TLR and Myd88 signaling cascade. In normal condition, senescent neutrophils die through spontaneous apoptosis in a short period of time. Longer survival of neutrophils due to inhibition of apoptosis contributes to the accumulation of these cells at inflammatory sites. During inflammation, cytokines including INFγ, IL-8, and IL-1β could result in longer neutrophil survival through inhibiting apoptosis^47^. In coherence with this, serum IFNγ levels were significantly higher in SCD mice which may be related to the increased bone marrow neutrophil senescence seen in SCD mice^11^. The research showed that signals derived from intestinal microbiome modulate neutrophil senescence in SCD mice^10^ and there was expansion of bone marrow senescent neutrophils, which was entirely abolished by microbiome depletion and was associated with dramatically improved osteoblast functions in co-culture^11^. The results from our current study, as well as our previously published data^11^ suggest the following concept: In SCD mice, increased bacteria load augments antigenic load traversing the impaired intestinal barrier through inflammation, leading to increased inflammatory cytokines and neutrophil aging, impaired osteoblast function, and low bone mass. Abx treatment partially rescued decreased bone mass due in part to decreased gut bacteria load.

One limitation of our study is that Abx treatment was only performed in male mice although we observed significantly reduced bone mass in both male and female^38^ SCD mice. Since gut bacterial load was also significantly higher in female SCD mice compared with Ctrl (data not shown), female SCD mice would be expected to have a similar response to Abx treatment. Another limitation of our study is that based on our previously published and current study data, we can only establish an association between neutrophil aging and bone loss in SCD mice. Future *in vitro* and *in vivo* studies using neutrophil senolytics to investigate the direct effect of neutrophil aging on bone loss are warranted. In addition, since our unpublished data shows that reduced bone mass in SCD mice is also associated with alterations in gut microbiome composition, the impact of different microbial communities on sickle bone disease needs to be investigated.

To our knowledge, this is the first report that decreased bone mass in SCD mice can be partially rescued by Abx-mediated microbiome depletion. Penicillin V has been widely used in clinics to prevent morbidity and mortality from Streptococcus *pneumonia* infection in young children with SCD^48–50^. The results from our current study raise the possibility that antibiotic therapy may mitigate sickle cell bone disease, which may warrant further evaluation in clinical studies.

## Methods

### Mice

Townes SCD mice^51^ were bought from The Jackson Laboratory (Bar Harbor, Maine, USA). These mice are on a mixed C57BL/6 and 129 genetic backgrounds. Townes SCD mice have both human α- and β-globin genes knocked into the mouse locus, allowing the generation of Ctrl (Homozygous for *Hba*^tm1 (HBA) Tow^, Homozygous for *Hbb*^tm3 (HBG1, HBB) Tow^) and SCD (Homozygous for *Hba*^*tm1 (HBA) Tow*^, Homozygous for *Hbb*^*tm2 (HBG1*, HBB*) Tow^) littermates by intercrossing sickle cell trait (SCT, Homozygous for *Hba*^tm1 (HBA) Tow^, Compound Heterozygous *Hbb*^tm2 (HBG1, HBB*) Tow/Hbbtm3 (HBG1, HBB) Tow^) mice. Ctrl and SCD littermates that was generated from mating SCT to SCT were used in the current study. The mice were hosted in the animal tower at the UConn Health. Mice were fed with Envigo Teklad Diet-2918 that contains 1% calcium, 0.7% total phosphate, and 1.5 IU D3/g of diet. All methods were performed in accordance with the relevant guidelines and regulations. All animal protocols were approved by the UConn Health Institutional Animal Care and Use Committee.

### Abx administration

Mice are coprophagic, therefore, to prevent gut microbiome exchange, Ctrl and SCD male mice were housed by genotype (genotype-housed) after weaning. To minimize cross-contamination and maintain individual microbiome, Ctrl and SCD male mice were caged individually (housed separately) at 4 months of age and were assigned to H_2_O or Abx groups randomly. The Abx administration regimens were adopted from Zhang *et al*.^10^. The treatment regimens are ampicillin, neomycin, metronidazole, and vancomycin in drinking water, all at 1 g/L. Drinking water or without Abx were change every 3 to 4 days. After 7 weeks treatment (at 6 months of age) mice were sacrificed using approved carbon dioxide protocol of euthanization for sample collection. Antibiotics were bought from Sigma (St. Louis, MO, USA).

### Dual beam X-ray absorptiometry (DEXA)

DEXA imaging was performed using the LunarPIXImus2 (GE Healthcare, Chicago, IL, USA) densitometer to measure BMD and BMC *in vivo* at the end of 7 weeks of treatment.

### μCT scanning of femurs

The mid-diaphysis cortical bones and metaphyseal cancellous bones of the distal femurs were used for analysis with μCT instrumentation (μCT20, Scanco Medical AG, Bassersdorf, Switzerland). Using two-dimensional data from scanned slices, three-dimensional analysis was conducted to calculate morphometric parameters defining micro-architecture, including cortical thickness, cortical area, periosteal perimeter, endosteal perimeter, cortical porosity, cortical apparent density, bone volume/total volume, trabecular number, trabecular thickness, trabecular spacing, trabecular apparent density, and structure model index.

### Bone histomorphometry

To analyze the bone turnover rate, intraperitoneal injections of calcein and xylenol orange were performed at 7 and 2 days, respectively, before sacrificing. Femurs were isolated and fixed in 10% formalin then placed overnight in 30% sucrose and embedded in Cryomatrix. Longitudinal central sections (7 μm) that include the central vein were collected on a cold adhesive tape, Cryofilm type IIC (FINETEC Co. Ltd., Japan). Unstained tapes with samples were soaked in PBS for half an hour and then mounted in 50% glycerol in PBS for dynamic parameter analysis. Additional sections were stained for tartrate-resistant acid phosphatase (TRAP) to visualize osteoclasts and counterstained with hematoxylin. Histomorphometric measurements were made in a blind, nonbiased manner using the OsteoMeasure image analysis system (R & M Biometrics, Nashville, TN, USA). The terminology and units used are those recommended by the Histomorphometry Nomenclature Committee of the American Society for Bone and Mineral Research^52^. Osteoclast number/bone surface, osteoclast surface/bone surface, osteoblast surface per bone surface, interlabel thickness, mineral apposition rate, mineral surface/bone surface, and bone formation rate/bone surface were measured. Osteoclasts were identified as multinucleated cells with more than three nuclei on the trabecular bone surface. Un-decalcified frozen sections were also used for von Kossa staining and Masson’s Trichrome staining.

### Serum biochemistry and cytokines

Serum was prepared from blood that was collected from euthanized animals by cardiac puncture. Serum calcium was measured using StanbioTotal Calcium LiquiColor® kit (StanBio Laboratory, Boerne, TX, USA). Serum phosphate was measured using the Stanbio Phosphorus Liqui-UV® kit (StanBio Laboratory, TX, USA). Mouse 1,25-Dihydroxy Vitamin D EIA kit and mouse C terminal Telopeptides of type l collagen (CTX-1) ELISA kit were purchased from Immuno Diagnostic Systems (Gaithersburg, MD, USA). Serum osteocalcin (OCN) was measured using mouse Osteocalcin ELISA Kit purchased from Immunotopics, Inc. (San Diego, CA, USA). Serum creatinine was measured using QuantiChrom Creatinine Assay Kit (BioAssay Systems, Highland, UT, USA). Serum inflammation cytokines IL10, INFγ, and IL27 were measured using Mouse Magnetic Luminex Assays kit (R&D Systems, Minneapolis, MN, USA).

### Real-time quantitative PCR (RT-qPCR) assay

The total RNA from flushed tibial shafts or the middle 20 mm of small intestines were isolated by utilizing Trizol reagent (Invitrogen, Carlsbad, CA, USA). The first-strand cDNA was synthetized using the Super-Script^TM^ First-Strand Synthesis Kit (Takara Bio USA Inc, Mountain View, CA, USA). RT-qPCR was performed using the iTaq™ Universal SYBR^®^ Green Supermix kit (BIO‐RAD Laboratories Inc., Hercules, CA, USA). The relative target gene expression was normalized to β-actin. The data were calculated using the method previously described^53^. The primers for the genes of interest are listed in Supplemental Table 1.

### Microbial DNA isolation from stool samples and RT-qPCR

Stool samples were obtained from the mice at 6 months of age (7 weeks post Abx administration). Samples were stored at −80 °C since collection until DNA extraction. One hundred mg stool sample from each mouse was used for microbial DNA isolation by utilizing the GenElute Stool DNA Isolation Kit (Sigma, St. Louis, MO, USA). The universal 16S rRNA primers (listed in Supplemental Table 1) were used for quantification of the amount of commensal bacteria by RT-qPCR.

### Statistics

Data are expressed as mean ± standard error (SE). Statistical significance (p < 0.05) was determined using IBM SPSS Statistics Version 20. The analysis of variance was performed using the post-hoc least significant difference test with the division into four groups according to genotype (Ctrl, SCD) and treatment (H_2_O, Abx).

The datasets generated during the current study are available from the corresponding authors on reasonable request.

## Supplementary information

Supplemental Table 1
