# Supplementary material for: Depletion of Intestinal Microbiome Partially Rescues Bone Loss in Sickle Cell Disease Male Mice
Source: Sci Rep. 2019 Jun 17;9:8659. doi: 10.1038/s41598-019-45270-4 (PMC6572770; doi:10.1038/s41598-019-45270-4)
Supplement: Supplementary file 1 — Supplemental Table 1 [file 41598_2019_45270_MOESM1_ESM.pdf]

Depletion of Intestinal Microbiome Partially Rescues Bone Loss in Sickle Cell Disease Male Mice

Sara Tavakoli[1], Liping Xiao*[1,2]

[1]Department of Medicine, [2]Department of Psychiatry, UConn Health, Farmington, CT, 06030, USA

**Supplemental Table 1. Primers used for RT-qPCR**		
**Gene**	**Forward**	**Reverse**
β-Actin	GTCGAGTCGCGTCCACC	CGCAGCGATATCGTCATCCA
Eubacteria	ACTCCTACGGGAGGCAGCAGT	ATTACCGCGGCTGCTGGC
Alp	GTGACTACCACTCGGGTGAAC	CTCTGGTGGCATCTCGTTATC
Col1a1	GGTCCTCGTGGTGCTGCT	ACCTTTGCCCCCTTCTTTG
Osterix	ACTGGCTAGGTGGTGGTCAG	GGTAGGGAGCTGGGTTAAGG
Runx2	GTTCAACGATCTGAGATTTGTG	GGGAGGATTTGTGAAGACTG
Ocn	GAGGGCAATAAGGTAGTGAACAGA	AAGCCATACTGGTTTGATAGCTCG
Rankl	CACCATCAGCTGAAGATAGT	CCAAGATCTCTAACATGACG
Opg	ATCCAAGACATTGACCTCTGTG	CTGTGGTGAGGTTCGAGTGG
Ctsk	GAAGAAGACTCACCAGAAGCAG	TCCAGGTTATGGGCAGAGATT
Igf1	CACATCATGTCGTCTTCACACC	GGAAGCAACACTCATCCACAATG
Tnfα	GACGTGGAAGTGGCAGAAGAG	TGCCACAAGCAGGAATGAGA
Il17	TGACGCCCACCTACAACATC	CATCATGCAGTTCCGTCAGC
Ifnγ	GGTCCAGCGCCAAGCAT	GCTGGATTCCGGCAACAG
Claudin3	TCATCACGGCGCAGATCA	CTCTGCACCACGCAGTTCA
Claudin15	GGCGGCATCTGTGTCTTCTC	TGGTGGCTGGTTCCTCCTT
